# Brillouin spectroscopy and radiography for assessment of viscoelastic and regenerative properties of mammalian bones

**DOI:** 10.1117/1.JBO.23.9.097004

**Published:** 2018-09-27

**Authors:** Dana Akilbekova, Vyacheslav Ogay, Talgat Yakupov, Madina Sarsenova, Bauyrzhan Umbayev, Asset Nurakhmetov, Kairat Tazhin, Vladislav V. Yakovlev, Zhandos N. Utegulov

**Affiliations:** a Nazarbayev University, National Laboratory Astana, Astana, Kazakhstan; b Nazarbayev University, School of Engineering, Department of Chemical Engineering, Astana, Kazakhstan; c National Center for Biotechnology, Stem Cell Laboratory, Astana, Kazakhstan; d Nazarbayev University, Department of Physics, School of Science and Technology, Astana, Kazakhstan; e Research Institute of Traumatology and Orthopedics, Astana, Kazakhstan; f Texas A&M University, Department of Biomedical Engineering and Department of Physics and Astronomy, College Station, Texas, United States

**Keywords:** Brillouin light scattering, bones, critical-sized defect, biomechanical properties, elastic, viscous, heparin-conjugated fibrin gel, bone morphogenic proteins, mesenchymal stem cells

## Abstract

Biomechanical properties of mammalian bones, such as strength, toughness, and plasticity, are essential for understanding how microscopic-scale mechanical features can link to macroscale bones’ strength and fracture resistance. We employ Brillouin light scattering (BLS) microspectroscopy for local assessment of elastic properties of bones under compression and the efficacy of the tissue engineering approach based on heparin-conjugated fibrin (HCF) hydrogels, bone morphogenic proteins, and osteogenic stem cells in the regeneration of the bone tissues. BLS is noninvasive and label-free modality for probing viscoelastic properties of tissues that can give information on structure-function properties of normal and pathological tissues. Results showed that MCS and BPMs are critically important for regeneration of elastic and viscous properties, respectively, HCF gels containing combination of all factors had the best effect with complete defect regeneration at week nine after the implantation of bone grafts and that the bones with fully consolidated fractures have higher values of elastic moduli compared with defective bones.

## 1 Introduction

Bone fractures are widespread, especially in the elderly population and people involved in extensive physical activity (e.g., sportsmen, military personnel, and heavy load physical labor force), and their impact is pervasive. From an individual’s perspective, a fracture negatively affects the physical and mental health, whereas complicated cases such as hip fractures increase can lead to the permanent disability and increased the risk of death. Direct and indirect costs of the fracture care can be high. According to the report on Osteoporosis in the European Union, in 2010 care expenditures of the healthcare systems for the fragility fractures were €37 billion.^1^

Autografts, allografts, and other bone grafts are current standard strategies for the bone fracture repair.^2^ However, integration of each bone graft substitutes can be limited due to the donor-site morbidity, risks of the infection, delayed healing, and others. Since last decade, work on the development of better bone graft substitutes has been at the forefront of medical research.^3^^–^^5^ Production of the clinically relevant bone grafts should ultimately have mesenchymal stem cells (MSCs) and growth factors embedded into the delivery material.^6^^,^^7^ MSCs isolated from periosteum have proven their efficiency in the regeneration of complex bone fractures.^8^ MSCs of periosteum show robust chondrogenesis and osteogenesis and induce the production of proangiogenic growth factors, stimulating the vascularization and accelerating the regeneration process.^9^^,^^10^ Stem cells can be differentiated into the osteoblasts using bone morphogenetic proteins (BMPs), such as BMP-2 and BMP-7. BMP-2 and BMP-7 are osteoinductive proteins that play an important role in the formation of new bone tissue.^11^ In this work, heparin-conjugated fibrin (HCF) hydrogel is used to deliver the both BMPs and stem cells.^12^ Bone repair was studied using critical size bone defects in the ulna of 12- to 16-week outbred rabbits.

To evaluate the efficacy of the bone grafts to regenerate the critical-sized defect, Brillouin light scattering (BLS) spectroscopy is utilized. Methods based on applying tension or compression loads to failure are commonly used for the biomechanical testing. These methods are useful for measuring bones from small animals. Noninvasive techniques, such as dual energy x-ray absorptiometry, peripheral quantitative computed tomography, and microcomputed tomography (μCT), are shown to be useful methods for quantitative assessment of bone strength.^13^ Above-mentioned methods have their own resolutions and by combining several methods, a full assessment of the bone’s mechanical parameters at various scales can be achieved. Bone has a hierarchically organized structure and mechanical properties vary from one structural level to another.^14^ For instance, at the macrolevel, the Young’s modulus of the cortical bone was measured to be around 20 GPs, whereas at the microlevel, it is 5.4 GPa.^15^ Mechanical properties of the cortical bones correlate with porosity, mineralization degree, and how solid matrix is organized; therefore, extrapolating mechanical properties at the microlevel (osteons, single trabeculae, and lamellae) and their fate *in vivo* from the mechanical parameters at the macrolevel can be a difficult task. Here, we aim to assess mechanical parameters of the cortical bone at the microlevel using BLS spectroscopy. It has proven to be a powerful noncontact, noninvasive, and label-free optical technique capable of assessing biomechanical (viscoelastic) properties at the micron-millimeter scale for a variety of biomedical applications.^16^^–^^19^ This technique is based on the inelastic scattering of laser light from hypersonic acoustic waves (phonons) in a material under study. The frequency shift of the incident light is proportional to the speed of sound in the medium and is called Brillouin frequency shift. Elastic modulus of the material is related to the speed of sound in the medium, and, therefore, by detecting the Brillouin frequency shift and measuring the velocities of acoustic waves, one can assess the elastic properties of the investigated material.^20^^,^^21^ The spectral linewidth of the Brillouin peak characterizes the extent of viscous (sound attenuation) behavior in the probed medium.^22^

For the past decades, the potential of the BLS techniques to study the mechanical properties of the biological tissues was demonstrated.^18^^,^^23^^–^^25^ BLS technique was used to distinguish between the malignant and the normal tissue.^21^ The mechanical contrast between normal and cancerous tissues can be used to detect and characterize the edge of the tumor.^26^^,^^27^ Advancements in the BLS instrumentation allows probing extracellular matrix and its proteins with high resolution and acquisition time.^17^^,^^28^^–^^31^ There are number of works reporting the biomechanics of the hard tissues.^32^^–^^37^ Bone healing at the implant site was investigated using micro-BLS technique and measured ultrasonic velocities in the newly formed bones and mature bones were in a good agreement with the histological analysis.^32^ The higher sound velocities, corresponding to the higher elastic modulus were observed for the mature bones having higher mineral content.^32^

The ultimate goal of all bone grafts is to decrease a time it takes to regenerate the critical-sized defect completely and to increase the bone healing quality.^12^ The quality of the implant integration and new bone formation can be assessed through the features, such as bone microstructure, elastic properties, and density of the newly formed bone.^34^ It is yet to understand what compositions of the bone grafts are needed to achieve the optimal mechanical and biological performance. Therefore, there is a need to develop approaches in the characterization of the biomechanical (viscoelastic) properties of newly formed bones and bone grafts efficacy that can eventually lead to better understanding of the bone regeneration processes and improving bone grafts performance. Atomic force microscopy and scanning acoustic microscopy are well-known contact techniques for viscoelasticity-specific imaging suitable for bone mechanics probing.^33^^,^^38^ Previously, efficacy of the injectable calcium phosphate bone substitute in the regeneration of the bone network was assessed using μCT imaging and nondestructive microindentation technique.^39^ These methods were shown to be relevant in performing quantitative bone morphometry measurements and results were well correlated with the results of the conventional mechanical compression test. However, a scrupulous specimen preparation, custom made setup, and complex image processing methods are major drawbacks of these methods. Therefore, Brillouin spectroscopy is an attractive noncontact technique for viscoelastic property assessment with a microscopic resolution around 10  μm and it can be easily incorporated with other optical modalities, such as Raman spectroscopy, optical coherent tomography, second harmonic generation imaging, and others.^28^^,^^36^^,^^40^^–^^42^

Most bone tissues are known to display viscoleastic, anisotropic, and nonlinear responses to an applied mechanical force. The study of viscoleasticity properties and load-dependent behavior of newly formed bones can provide insights into the regeneration efficacy of the bone defects. Previously, mechanical tests under a single load showed that that the mineral density and collagen organization play an important role in the bone strength, toughness, and eventually in failure mechanisms.^43^ The primary focus of our research study was to investigate the relationship between the compression load and mechanical strength of various mammal bones (bovine, ovine, and chicken tibia) determined from measured Brillouin frequency shifts, linewidths, and the bone regeneration efficacy of the critically sized defects in rabbits ulna implanted with bone grafts (HCF gels, BMPs, and osteogenic stem cells).^44^

## 2 Methods

### 2.1 Bone Specimen Preparation and Ethics Statement

**Fig. 1:**
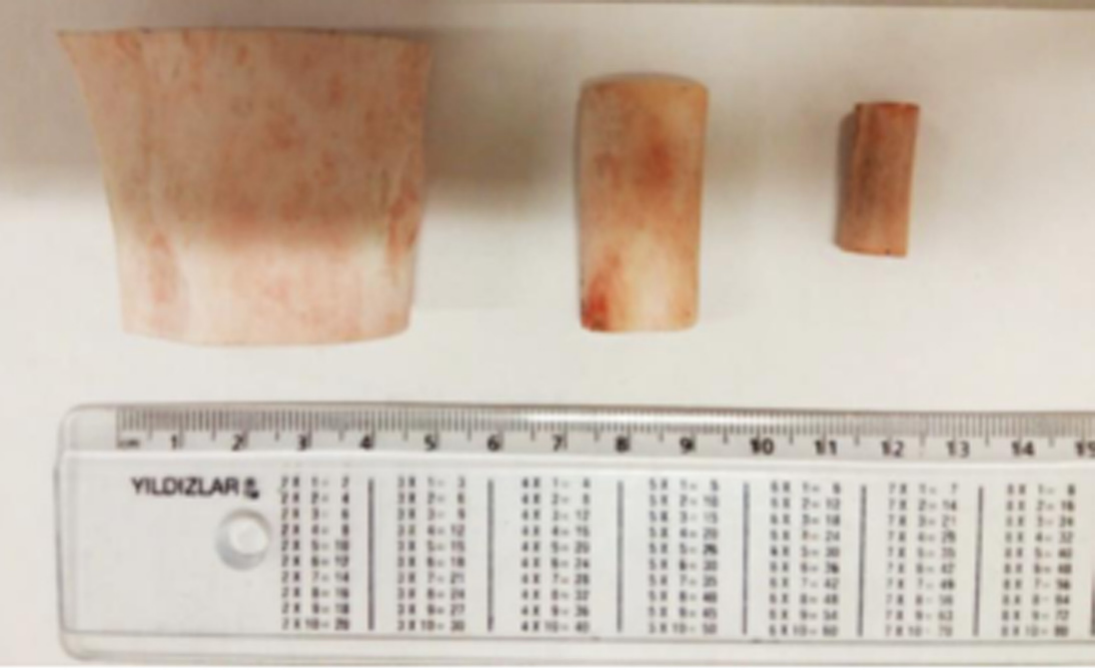
Photographs of the bone samples. Bovine, ovine, and chicken tibia bones.

Cortical bones of different mammalian species and rabbit critical-sized bone defect model were used. Bone regeneration was studied using critical size bone defects in the ulna of 12- to 16-week-old outbred rabbits. The research protocol was approved by the local animal ethics committee at National Laboratory Astana prior the animal study. Bovine, ovine, and chicken tibia bones were cleaned and cut into small pieces along the radial plane (Fig. 1) and were polished to achieve the smooth and even surface.

### 2.2 Preparation of Bone Grafts

First, MSCs were derived from the periosteum of rabbits. Samples were isolated from the proximal tibial epimetaphysis under general anesthesia (ketamine 5  mg/kg intramuscularly) and then obtained periosteum was minced to the small pieces and treated with a solution of type I collagenase overnight (16 to 18 h) to completely dissociate the tissue. Isolated periosteal cells were washed with phosphate buffered saline (PBS) and resuspended in the complete α-MEM culture medium containing 10% fetal bovine serum, 2 mM L-glutamine, 100  U/mL penicillin, 100  μg/mL streptomycin, and 0.25  μg/mL amphotericin B (all from Life Technologies). Next, HCF hydrogel was obtained. In the first step, 100 mg of heparin (5000 Da) was completely dissolved in 1 mL of a buffer of 2-(N-morpholino) ethanesulfonic acid (0.05 M, pH 6.0). Then, 0.0046 g of N-hydroxysuccinimide (NHS) and 0.0153 g of N-(3-dimethylaminopropyl)-N-ethylcarbodiimide hydrochloride (EDC) were added. Solution was precipitated using acetone and lyophilized at −20°C for 24 h. For the conjugation of activated heparin with fibrinogen, a lyophilized NHS solution of heparin (60 mg) was dissolved in 20 mL of PBS (pH 7.4), and then conjugated with fibrinogen (100 mg) for 3 h at 4°C. The resulting solution was precipitated with acetone and lyophilized.

To obtain various combinations of HCF grafts, MSCs of rabbit periosteum ($2\times 10^{6}\text{ cells}$) and/or osteoinductive factor BMP-2 (400  ng/mL) were mixed with 50  U/mL thrombin and 250  U/mL aprotinin in a volume of 400  μL. Then, HCF (40  mg/mL) and fibrinogen (40  mg/mL) were dissolved in the α-MEM nutrient medium (400  μL) and added in equal volume to the solution containing the MSCs, thrombin, and aprotinin. After mixing all the components, hydrogels with cells or with growth factors were implanted into the bone defects.

### 2.3 Defect formation and bone graft implantation

**Fig. 2:**
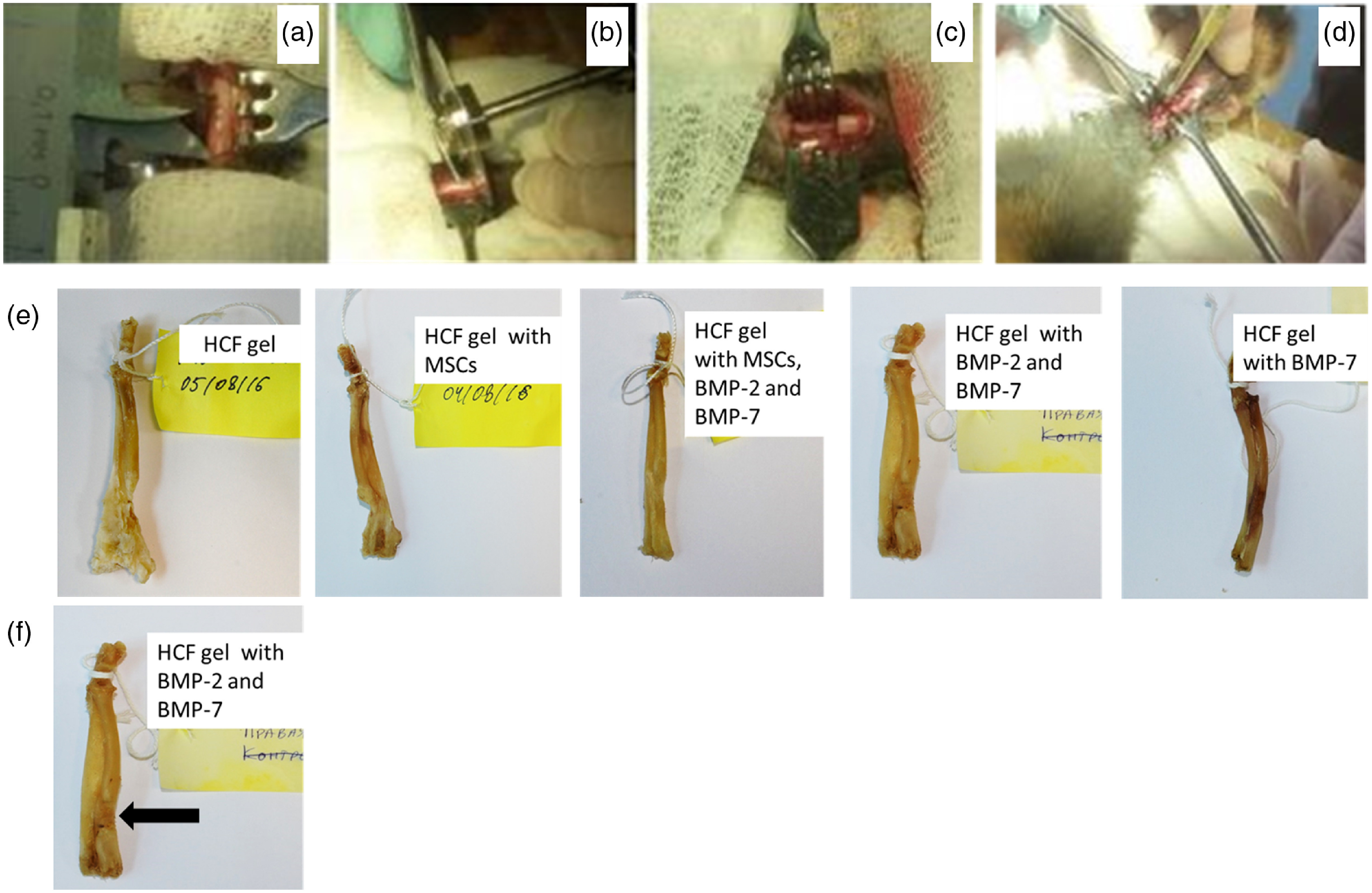
Photographs of the surgical procedures and ulna bones of the rabbits. (a) Section of the radius before resection, (b) resection of the bone with an oscillating saw, (c) created critical-sized defect, (d) implantation of the bone grafts with various combinations of the HCF gels, MSCs and BMPs into the defect area, (e) ulna bones of the rabbits after the sacrifice, and (f) location of the point-measurement spot on the ulna bone.

Full thickness segmental critical-sized defects (length ∼1.0  cm) were created on a long tubular bone on the diaphyseal part of the radius in the lower third of the forearm under the general anesthesia (ketamine 5  mg/kg intramuscularly) using a surgical oscillator saw. Then, HCF gels (400  μL) with various combinations of the components, such as BMP-2 and BMP-7 (400  ng/mL) and MSCs ($2\times 10^{6}\text{ cells}$) were injected into the defects of the control and test groups. Once gelation completed, the surgical wound was sutured. Bone defect regeneration was evaluated every 3 weeks using a mobile x-ray equipment (Practix 160, Philips). After 12 weeks, animals were sacrificed and the implanted ulnas were removed (Fig. 2). In the scope of this work, the effect of the gel alone versus the defect regeneration without any treatment was not studied.

### 2.4 Radiography and Computer Tomography of Bone Regeneration

The dynamics of the bone defect regeneration after the implantation of the fibrin hydrogel with periosteum cells and growth factors was recorded using radiography (Practix 160, Philips) every 3, 6, and 9 weeks.

Bone density after the sacrifice was evaluated using μCT (IVIS Spectrum CT; Caliper) with 150  μ voxel size, 440 Al fitter, 50 kV, resolution 425, and field of view L×W×H 12×12×13  cm. The approximate dose was 52 mGv per scan. The three-dimensional (3-D) reconstruction and bone density assessment were performed using the Living Image 4.3.1 software (Caliper). The acquired image was exported in the DICOM format and stored. Bone density was evaluated using Hounsfield units (HU). This unit is a standardized CT attenuation coefficient and represents the relative mass density of tissues, where for air this value is −1000  HU and water 0 HU.^45^

### 2.5 BLS Spectroscopy Measurements

**Fig. 3:**
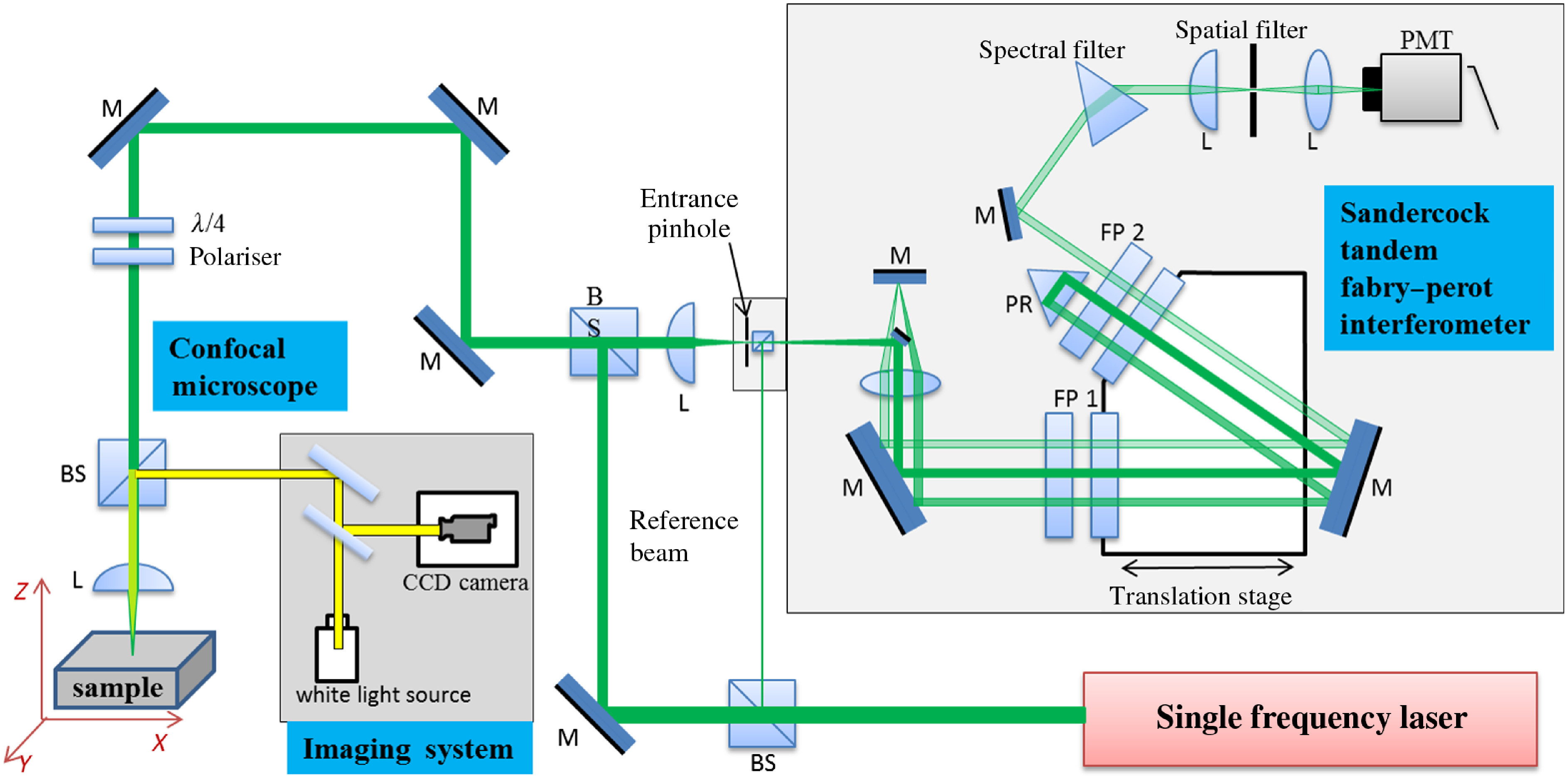
Schematic representation of the confocal BLS spectroscopy setup. Tandem Fabry–Perot interferometry coupled to confocal microscopy for spontaneous Brillouin microspectroscopy.

Incident laser beam from a single longitudinal mode 532-nm solid-state laser (Coherent Verdi G2) was focused on bone samples using confocal microscope via 20× objective (Mitutoyo, WD=20  mm, NA=0.42). The average optical power at the samples was kept at 10 to 15 mW (Fig. 3). Tandem Fabry–Perot interferometer equipped, coupled with a single-photon counting photomultiplier tube was used to collect BLS spectra in a backscattering geometry (TFP-1, JRS Scientific Instruments).^19^ Measurements were performed at the compressive loads along the bones’ axial direction with 2 and 10 kg incremental steps using the force gage (PCE-FM1000, PCE Instruments). Free spectral range of 35 GHz and ∼5  min per spectrum was used for each measurement. Repeated measurements were performed on the same bone in the same location. Incident laser beam spot was focused on the graft implantation site containing newly formed bone. Location of the spot is shown in Fig. 2(f). Acoustic wave velocities under elasto-optic Brillouin scattering mechanism were determined according to the following equations:^17^^,^^21^

$$v=f\frac{\lambda_{0}}{2n\;\cos\;\frac{\theta }{2}},$$ (1)

where f is the measured Brillouin frequency shift, n is the refractive index of a sample, $\lambda_{0}$ is the wavelength of the incident laser in air, and θ is the angle between the incident and scattered light directions, respectively. The value of the bone’s refractive index was ∼1.57 and assumed to be constant for all investigated bone samples.^46^ The Brillouin linewidth characterizes the attenuation of the sound wave and will be controlled by the viscosity and the Brillouin linewidth is given as^19^^,^^47^

$$\Gamma_{B}=\frac{q^{2}}{2\rho_{o}}(\frac{4}{3}\eta_{S}+\eta_{v}),$$ (2)

where $\eta_{S}$ and $\eta_{d}$ are the shear and volume viscosity coefficients, respectively, $q=(\frac{4\pi n}{\lambda })\sin(\frac{\Theta }{2})$ is the wave vector magnitude and $\rho_{0}$ is the average density of the material.

## 3 Results and Discussion

**Fig. 4:**
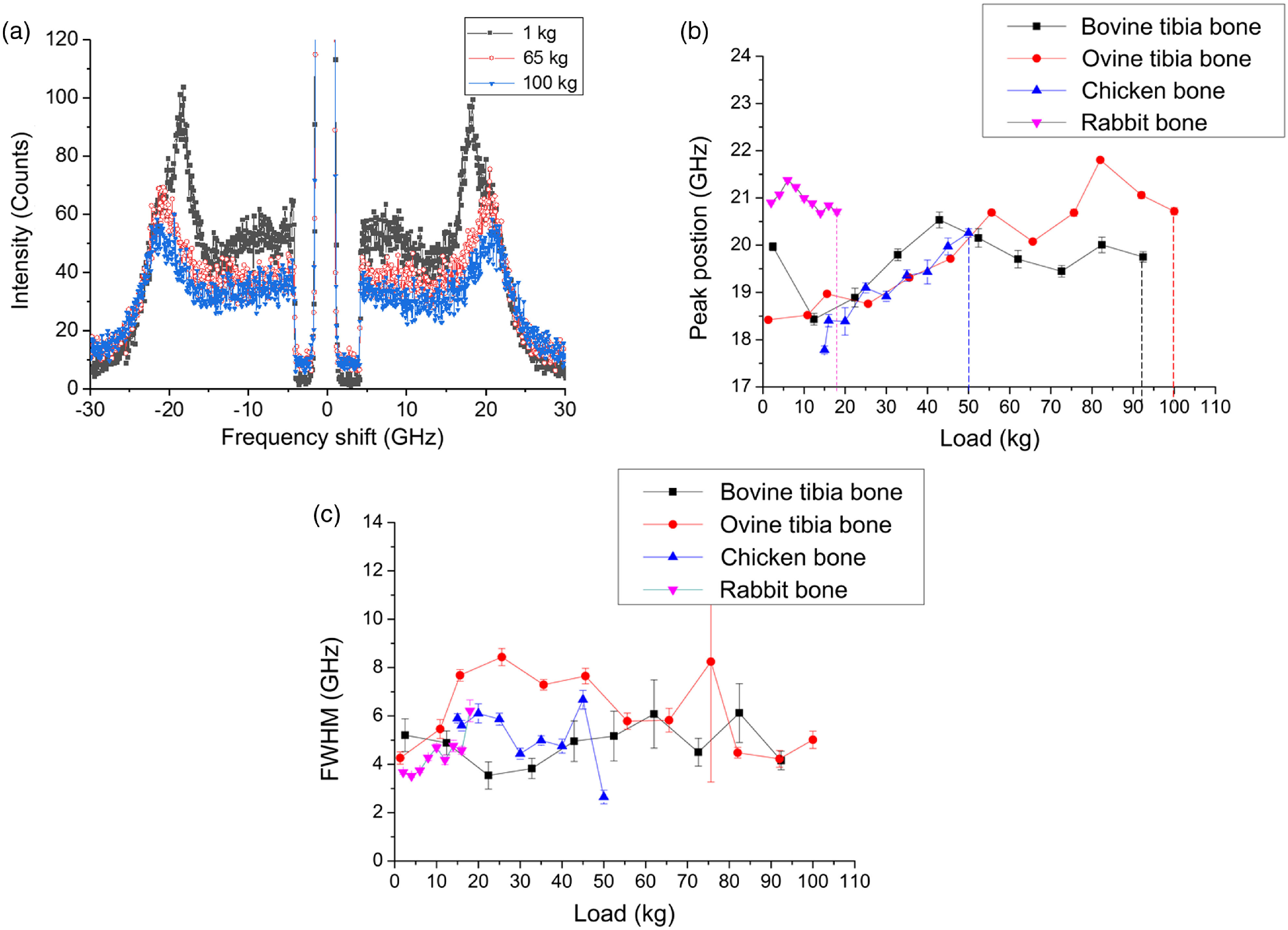
(a) Brillouin spectra acquired for the ovine tibia bone, (b) the Brillouin peak’ positions for bovine, ovine, chicken tibia, and ulna bones under the compression load. Error bars represent a 95% confidence interval based on the standard deviation. Corresponding breaking limits are shown with vertical dashed lines, and (c) FWHM obtained from the spectra as a function of compression load for bovine, ovine, chicken tibia, and rabbit ulna bones.

First, we measured the Brillouin shift of the bovine, ovine, chicken tibia bones, and rabbit ulna under the increasing compression load. Obtained spectra from the bone samples are in a good agreement with those reported in the literature.^35^^–^^37^^,^^40^ Figure 4(a) shows examples of Brillouin spectra of the ovine’s bone acquired at compressive loads up to 100 kg. Each spectrum corresponds to a specific compression load. From Fig. 4(a) Brillouin peaks, both the Stokes and anti-Stokes components can be distinguished. The Brillouin peak positions were plotted against the load applied to bone specimens of all animals [Fig. 4(b)]. The position of these peaks was different for each of the shown spectra. For instance, the Brillouin frequency shifts were 18.42±0.05  GHz (1 kg) and 20.72±0.07  GHz (100 kg) for ovine bone, corresponding to around 400  m/s difference with n=1.6 for the calcified matrix.^17^^,^^21^^,^^48^ Ovine tibia, bovine tibia, chicken bone, and rabbit bone broke at 100-, 93-, 50-, and 18-kg loads, respectively. Corresponding breaking limits of the bones are shown in Fig. 4(b) by vertical dashed lines. We can clearly see that the magnitude of the bones strength increased from rabbit to ovine, as expected. This difference occurs due to the different types of bones we used such as ovine, bovine, and chicken tibia bones, whereas rabbit bone was ulna. Rabbit long bones generally consist of primary bone tissue (less-organized collagen and lower bone density), whereas sheep shows a significantly higher density than a human and therefore greater strength. Bovine bones are described with the largely secondary bone (well-organized collagen and large osteon sizes).^49^^–^^51^ Overall composition and architecture of the bone tissue determine the mechanical properties of the cortical bone. With an increase in the applied compressive load, the observed Brillouin peak frequency shift increased for all bones suggesting an increased stiffness of the material and then slight decrease in the peaks was observed with a further increase in the load (stiffness degradation).^17^ Obtained results suggest that acoustic wave velocities (i.e., the elastic moduli) are affected by the compression load and BLS spectroscopy can be utilized to measure these changes. Additionally, spectral linewidth defined as the full width at half maximum (FWHM) of the Brillouin peak, was determined for studied bones [Fig. 4(c)]. These linewidths are signature of high-frequency viscous behavior of the bone medium—the larger the linewidth the more viscous is the bone medium, i.e., there is more hypersound attenuation and can be helpful in understanding the quality of the bone matrix, particularly degree of the mineralization.^22^^,^^37^^,^^52^ Recently, it was demonstrated that mineral phase controls the viscoelastic properties of the bone matrix.^53^

**Fig. 5:**
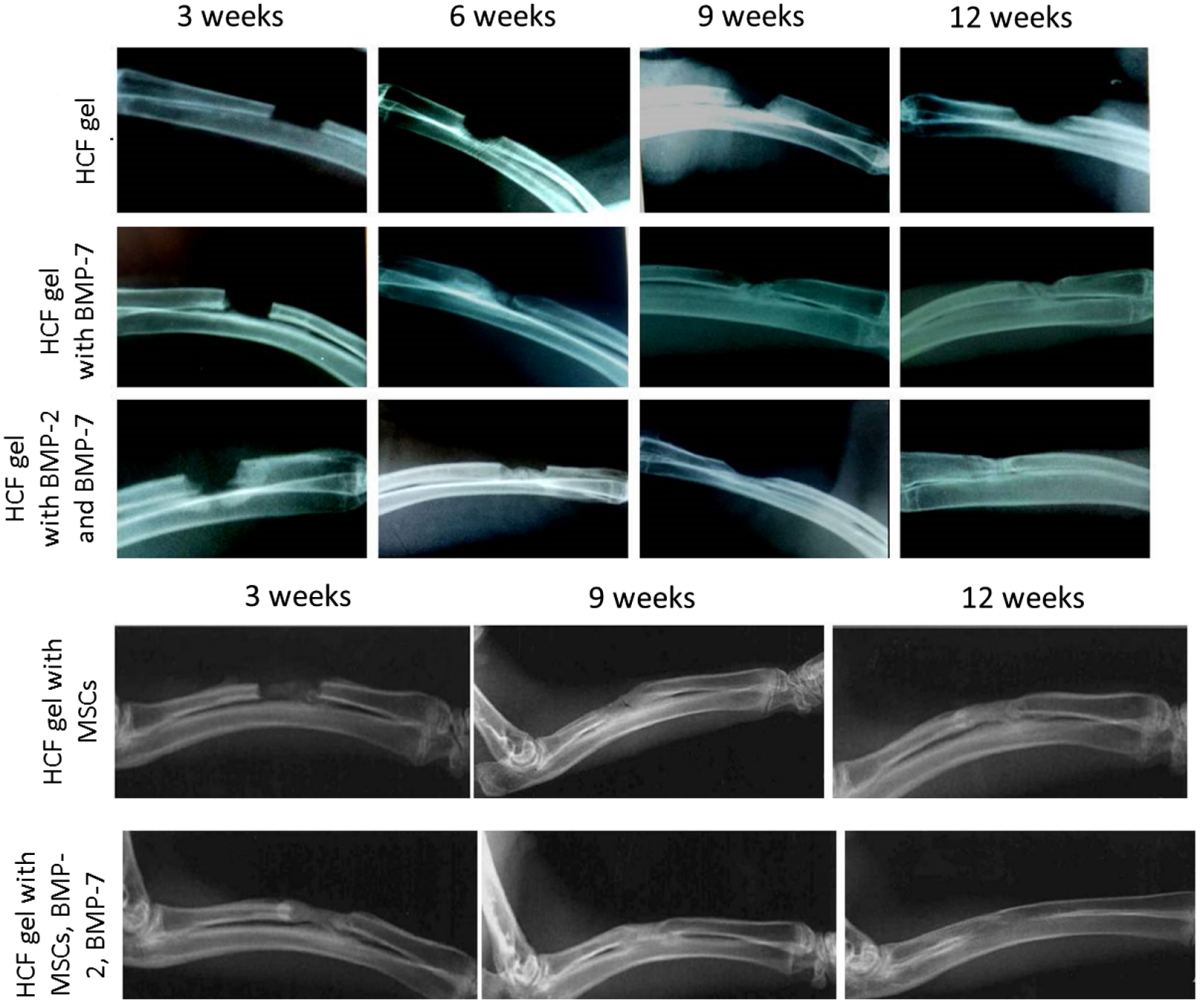
Dynamics of the bone defect regeneration after HCF implantation with MSCs, BMP-2, and BMP-7.

Regeneration of critical-sized defects in the rabbit ulnas was evaluated every 3 weeks. Corresponding x-ray images are shown in Fig. 5. Those images show that control samples with just HCF gel were not sufficient to heal the defect after the implantation. Healing of the critical-sized defects occurs through the development of the periosteal callus that bridges the fracture edges.^54^ Accelerated bone healing correlates with an increased callus formation contributing to the improved biomechanical stiffness.^55^ After three weeks, experimental groups implanted with gels containing BMPs showed similar regeneration dynamics with the formation of the periosteal callus in all groups. At week six, the formation of endosteal callus in the bone defects was observed. During the endosteal callus formation, fracture line disappears and becomes less visible in the radiographs. Notable changes were observed at week nine, the formation of an intermediate corn and a cortical layer, leading to a splicing of the defect. After 12 weeks, a virtually complete regeneration of the osseous cavities was observed.

Complete closure in the defect areas was already observed after 9 weeks for the experimental groups with bone grafts containing the combination of stem cells and all BMP, whereas in the group with only stems cells the formation of endosteal callus was observed and complete regeneration was observed later at week 12.

**Fig. 6:**
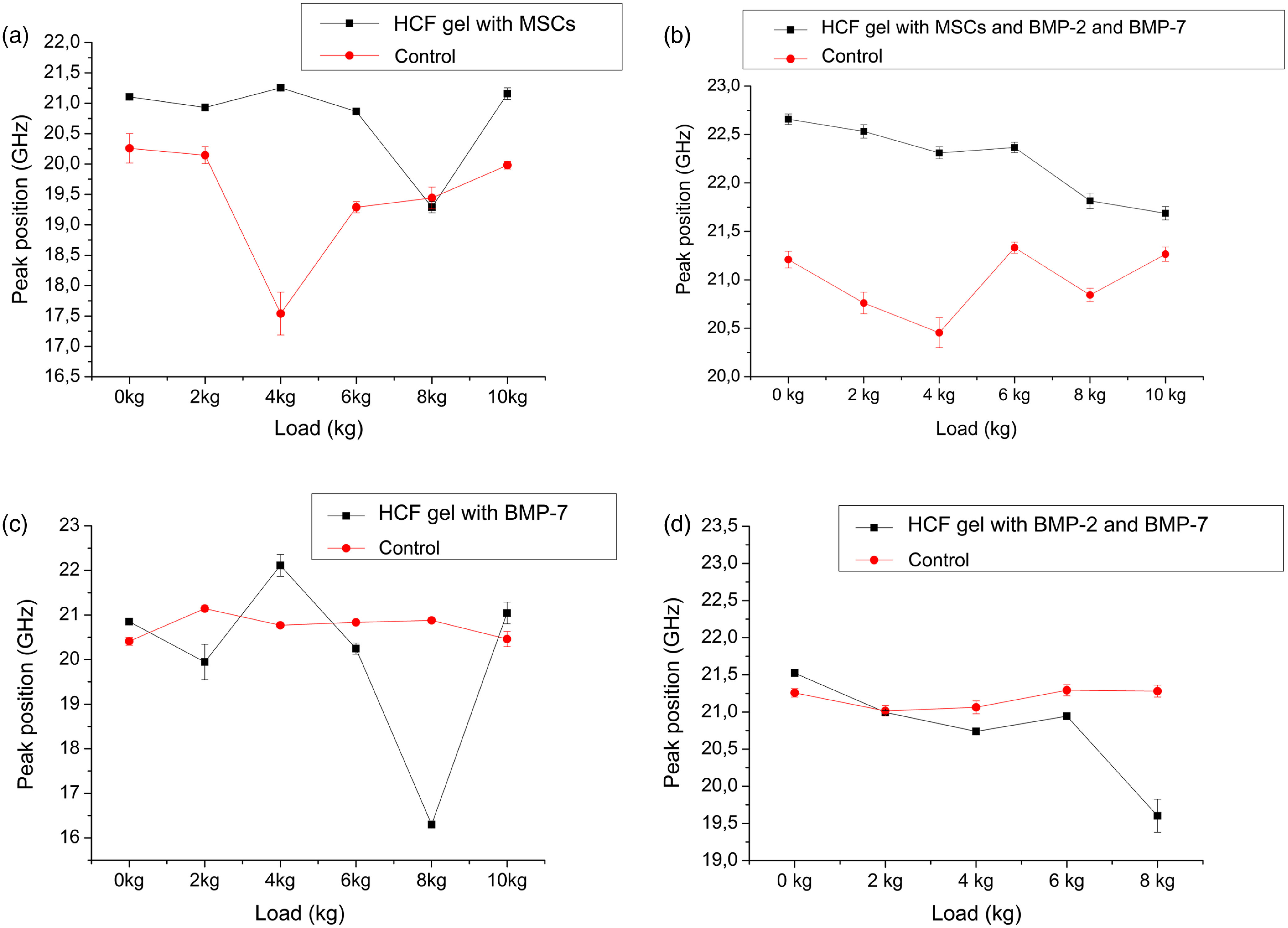
The Brillouin peaks obtained from the spectra as a function of compression load for the different bone grafts (a) HCF gel with MSCs, (b) HCF gel with MSCs, BMP-2, and BMP-7, (c) HCF gel with BMP-7, and (d) HCF gel with BMP-2 and BMP-7.

Next, we measured Brillouin spectra for the experimental groups with the grafts consisting of the HCF gel with different combinations of the stem cells and BMPs. Additionally, for each group, separate control with only HCF was tested. Compression experiments were performed to test the bone strength and BLS measurements were done simultaneously as a load was applied. With an increase in the compression load, significant variations in the Brillouin frequency shift was observed compared with the control samples with HCF gels only [Figs. 6(a) and 6(b)], dropping from 20.25±0.24  GHz to 17.54±0.35  GHz at the compression load of 4 kg and returning to the 19.28±0.091  GHz at the compression load of 6 kg. Similar variations were observed for the bones with the HCF gel with BMP-7, suggesting that these bones were not completely regenerated and there were some small defects and empty spaces left in the defect as seen in the x-ray images of these bones. Bones implanted with HCF gels consisting of stem cells and BMPs [Fig. 6(b)] and only BMPs [Fig. 6(d)] exhibited the decrease in the Brillouin frequency shift with the increased compression load. For all loads, MSC addition appears to be critically important for bone strength with or without BMPs compared with control samples, as shown in Fig. 6. Importantly, bones with implanted bone grafts in the series of “HCF gel with BMP-2 and BMP-7”—“HCF gel with BMP-7”—“HCF gel with MCS”—“HCF gel with MCS, BMP-2 and BMP-7” tend to get stronger, as evidenced from corresponding Figs. 6(a)–6(d), which is also supported by radiography images shown in Fig. 5.

In native bones the observed Brillouin peak frequency shift increased with a compression load [Fig. 4(b)], whereas in the experimental bones these peaks either decreased or stayed the same. Previously, it was shown that bones exhibit a stress-dependent nonlinear viscoelastic response. Bone samples become stiffer and softer with an increase in the stress. This effect was hypothesized to be due to the reorganization of the microstructural components (collagen fibrils) and degree of the mineralization of these fibrils. The bone matrix becomes stiffer in the beginning under the load and then localized buckling and accumulation of the microdamages cause softening and eventually can lead to the failure.^56^ Our results suggest that in the grafted samples, there are still nonmineralized collagens; therefore, at a higher load their elastic modulus decreases (higher deformability), whereas in native bones mineralized collagen fibrils elastic modulus increases with a load.^57^ Additionally, location of the measurement spot significantly impacts the ultrasonic velocity in the bone graft samples, where newly formed bone is located in the center of the graft implantation site surrounded by the mature bone.^32^ Therefore, one can expect the increase in the ultrasonic velocity or elastic modulus when moving away from the bone graft interface.^58^^,^^59^

**Fig. 7:**
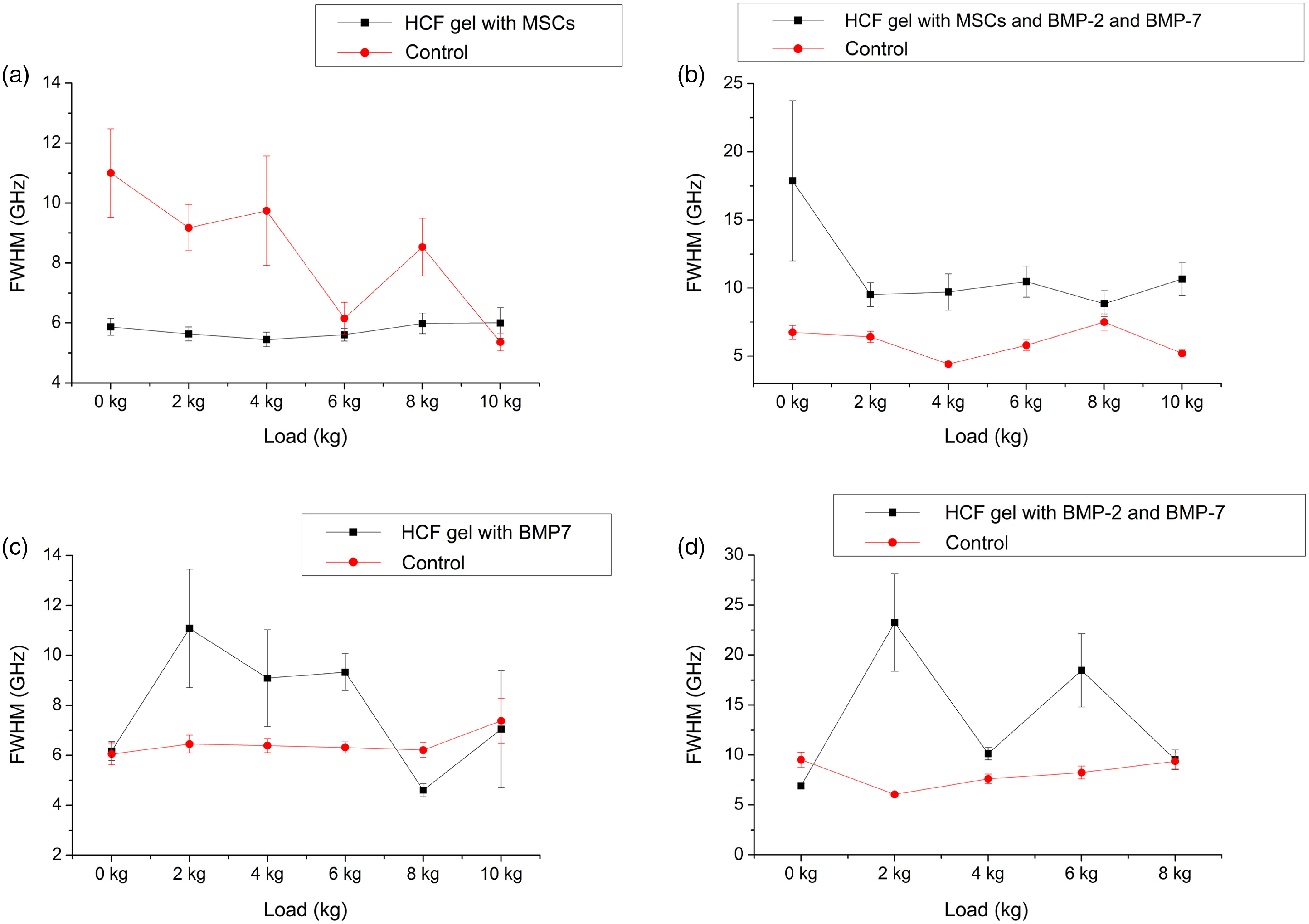
FWHM obtained from the spectra as a function of compression load for the different bone grafts (a) HCF gel with MSCs, (b) HCF gel with MSCs, BMP-2, and BMP-7, (c) HCF gel with BMP-7, and (d) HCF gel with BMP-2 and BMP-7.

Furthermore, FWHM values of the Brillouin peaks of investigated bones were also determined (Fig. 7). Addition of BMPs is the cause for the bones to be more viscous compared with control samples, as evidenced from Figs. 7(a)–7(d). Figure 7 shows FWHM values for all bones samples under the compression load. According to the results, the variation in FWHM under the load was noticeable for the bones with HCF gel and BMPs with no MSCs [Figs. 7(c) and 7(d)], whereas bones regenerated with gels containing MSCs show little or no change in the FWHM values under compression [Figs. 7(a) and 7(b)].

In solid materials, viscosity is a measure of the viscous-like deformation; materials with lower degree of viscosity can withstand more viscous-like deformation under constant loading.^60^ In newly formed bones, organic component (collagen) plays an important role in defining the viscoelastic properties of the bone and during the mineralization stage as mineral content increases mechanical stability grows as well.^61^ Taking together, the correlation between elastic modulus and viscous properties (i.e., Brillouin peak shifts and linewidths) of the regenerated bone graft with its initial composition (with or without MSCs or BMPs) can be interpreted as the variation in the mineral and organic portions of the bone matrix.^62^ Our results suggest that BLS can be sensitive to these variations.

Here, measured Brillouin spectra were used to determine hypersonic longitudinal sound wave velocities to compare elastic properties of regenerated bones. The acoustic sound velocities in the control bones were found to be in the range of $(3.43\text{ to }3.60)\times 10^{3}\;m/s$, whereas in the experimental groups velocity values were $(3.53\text{ to }3.84)\times 10^{3}\;m/s$ with the highest value for the HCF gel with MSCs, BMP-2, and BMP-7 (Table 1). These values are in a good agreement with the previously reported data.^21^^,^^33^ The literature values for the mature and newly formed bones of rabbit tibiae are $(4.81\text{ to }5.22)\times 10^{3}$ and $(4.83\text{ to }5.00)\times 10^{3}\;m/s$, respectively.^21^ It can be understood from a simple fact that, in the newly formed bones, the degree of mineralization is lower compared with the mature bones, leading to the lower values of the acoustic wave velocities (the elastic moduli).^17^^,^^32^ Some discrepancy in those values are, most probably, due to the fact that the tensile strength of ulna is lower than tibia bones, which undergo more load than the upper extremities. We compared values of the bone density from μCT measurements with acoustic velocities. From Table 1, we can clearly see that bone densities under no compressive load with the combination of HCF gel, MSCs, BMP-2, and BMP-7 (1550 HU) scales with corresponding acoustic velocities, which implies that elastic modulus is highest for this bone having highest degree of regeneration. Additionally, this sample displayed highest viscous property, as evidenced from highest Brillouin linewidth, suggesting an increase of the mineral content in the bone matrix.^62^

**Table 1:** Longitudinal acoustic wave velocities and mass densities of bone grafts.

	Acoustic velocity ($\times 10^{3}\;m/s$) at 0 kg	FWHM (GHz)	Bone density HU
HCF gel with MSCs	3.58±0.01	5.89±0.28	1170
Control	3.43±0.04	10.89±1.48	911
HCF gel with MSCs, BMP-2, and BMP-7	3.84±0.01	17.86±5.88	1550
Control	3.59±0.01	6.75±0.5	974
HCF gel with BMP-2 and BMP-7	3.65±0.01	6.91±0.24	1330
Control	3.60±0.01	9.52+0.76	1250
HCF gel with BMP-7	3.53±0.01	6.17±0.38	1130
Control	3.46±0.01	6.06±0.44	1560

In line with the previous studies, where HCF gels with stem cells and various growth factors were successfully used for the bone regeneration purposes, we showed the effectiveness of the MSCs combined with both BMPs.^12^^,^^63^^,^^64^

The sensitivity of the wave velocities to the changes in macro- and microstructural components in the bone matrix demonstrates extremely promising avenues for further employment of the BLS technique for tissue engineering applications in clinical settings for studying the quality of the bone grafts implantations through the characterization of the collagen fibrils, mineral content, and mechanical stability of the bone grafts itself and how different external parameters affect the regeneration process (temperature, pressure, and others). Introducing fiber optics can enable a real-time monitoring and incorporation of the BLS system into the other imaging setups.

Traditional Brillouin spectroscopy setups take point measurements and long-time measurements of a weak Brillouin scattering light are required (acquisition time about 100 ms).^35^^,^^65^ To overcome this problem and perform two-dimensional BLS measurements, several configurations are proposed such as introduction of a coherent phonon technique, virtually imaged phased array based either on stimulated or spontaneous Brillouin scattering.^22^^,^^65^^,^^66^ The recent Brillouin microscopy setup designed by Zhang et al.^65^ is based on spontaneous Brillouin scattering and enables simultaneous measurement of the hundreds of points in a sample with an acquisition time of <1  ms per point and spatial resolution 3.29-μm per pixel close to diffraction limit.

Our work also has a few limitations, performing series compressive creep-recovery experiments can help to understand more time-dependent viscoelastic behavior of the regenerated bone matrix. Elaborate preparation process of the samples suitable for the BLS studies and long-time measurements are the future challenges of the BLS systems and can hinder its application in clinical setting.

## 4 Conclusion

In this work, we evaluated viscoelastic properties of the mammalian bones and the regeneration efficiency of the critical-sized defects in rabbit ulna using BLS spectroscopy and radiography methods. The bovine tibia showed superior fracture resistance compared with the ovine and chicken bones. The reported study also revealed that HCF gels containing different combinations of the stem cells and BMPs were most effective in bone regeneration, whereas a combination of all factors had the best effect with complete defect regeneration after 9 weeks. Variations in the Brillouin shifts under the compression load were observed for the bones, where defects were not able to completely regenerate. Obtained results indicate that the bones with fully consolidated fractures have higher values of the acoustic wave velocities relating to higher elastic moduli compared with the bones with defects. The addition of MCS and BPMs is critically important for the regeneration of elastic and viscous properties, respectively. Micro-CT and radiography images are in good agreement with BLS characterization. BLS spectroscopy (for sensing of hypersonic velocity and sound attenuation) in tandem with radiography (for imaging and mass density determination) is also complimentary for full viscoelastic property assessment. These findings demonstrate extremely promising avenues for further employment of BLS technique in combination with radiography for assessment of strength and viscous properties of large variation of viscoelastic strength and for tissue engineering applications.
